# RNA-binding protein GIGYF2 orchestrates hepatic insulin resistance through STAU1/PTEN-mediated disruption of the PI3K/AKT signaling cascade

**DOI:** 10.1186/s10020-024-00889-6

**Published:** 2024-08-13

**Authors:** Ziwei Lv, Yuanyuan Ren, Yang Li, Fanglin Niu, Zhuozhuo Li, Man Li, Xiaofang Li, Qinhua Li, Deqing Huang, Yi Yu, Yuyan Xiong, Lu Qian

**Affiliations:** 1 Key Laboratory of Resource Biology and Biotechnology in Western China, Ministry of Education, Faculty of Life Sciences and Medicine, College of Life Sciences, Northwest University, 229 Taibai North Road, Xi’an, 710069 Shaanxi P.R. China; 2 Shaanxi Key Laboratory of Brain Disorders & Institute of Basic and Translational Medicine, Xi’an Medical University, Xi’an, 710018 Shaanxi P.R. China; 3 Department of Endocrinology, The Affiliated Hospital of Northwest University, Xi’ an No.3 Hospital, Xi’an, 710018 Shaanxi P.R. China; 4 Department of Gastroenterology, The Affiliated Hospital of Northwest University, Xi’ an No.3 Hospital, Xi’an, 710018 Shaanxi P.R. China; 5 Xi’an Mental Health Center, Xi’an, 710100 Shaanxi P.R. China; 6 Xi’an Key Laboratory of Cardiovascular and Cerebrovascular Diseases, The Affiliated Hospital of Northwest University, Xi’an No.3 Hospital, Xi’an, 710018 Shaanxi P.R. China

**Keywords:** GIGYF2, Insulin resistance, STAU1, RNA-binding protein

## Abstract

**Background:**

Obesity is well-established as a significant contributor to the development of insulin resistance (IR) and diabetes, partially due to elevated plasma saturated free fatty acids like palmitic acid (PA). Grb10-interacting GYF Protein 2 (GIGYF2), an RNA-binding protein, is widely expressed in various tissues including the liver, and has been implicated in diabetes-induced cognitive impairment. Whereas, its role in obesity-related IR remains uninvestigated.

**Methods:**

In this study, we employed palmitic acid (PA) exposure to establish an in vitro IR model in the human liver cancer cell line HepG2 with high-dose chronic PA treatment. The cells were stained with fluorescent dye 2-NBDG to evaluate cell glucose uptake. The mRNA expression levels of genes were determined by real-time qRT-PCR (RT-qPCR). Western blotting was employed to examine the protein expression levels. The RNA immunoprecipitation (RIP) was used to investigate the binding between protein and mRNA. Lentivirus-mediated gene knockdown and overexpression were employed for gene manipulation. In mice, an IR model induced by a high-fat diet (HFD) was established to validate the role and action mechanisms of GIGYF2 in the modulation of HFD-induced IR in vivo.

**Results:**

In hepatocytes, high levels of PA exposure strongly trigger the occurrence of hepatic IR evidenced by reduced glucose uptake and elevated extracellular glucose content, which is remarkably accompanied by up-regulation of GIGYF2. Silencing GIGYF2 ameliorated PA-induced IR and enhanced glucose uptake. Conversely, GIGYF2 overexpression promoted IR, PTEN upregulation, and AKT inactivation. Additionally, PA-induced hepatic IR caused a notable increase in STAU1, which was prevented by depleting GIGYF2. Notably, silencing STAU1 prevented GIGYF2-induced PTEN upregulation, PI3K/AKT pathway inactivation, and IR. STAU1 was found to stabilize PTEN mRNA by binding to its 3’UTR. In liver cells, tocopherol treatment inhibits GIGYF2 expression and mitigates PA-induced IR. In the in vivo mice model, GIGYF2 knockdown and tocopherol administration alleviate high-fat diet (HFD)-induced glucose intolerance and IR, along with the suppression of STAU1/PTEN and restoration of PI3K/AKT signaling.

**Conclusions:**

Our study discloses that GIGYF2 mediates obesity-related IR by disrupting the PI3K/AKT signaling axis through the up-regulation of STAU1/PTEN. Targeting GIGYF2 may offer a potential strategy for treating obesity-related metabolic diseases, including type 2 diabetes.

**Graphical Abstract:**

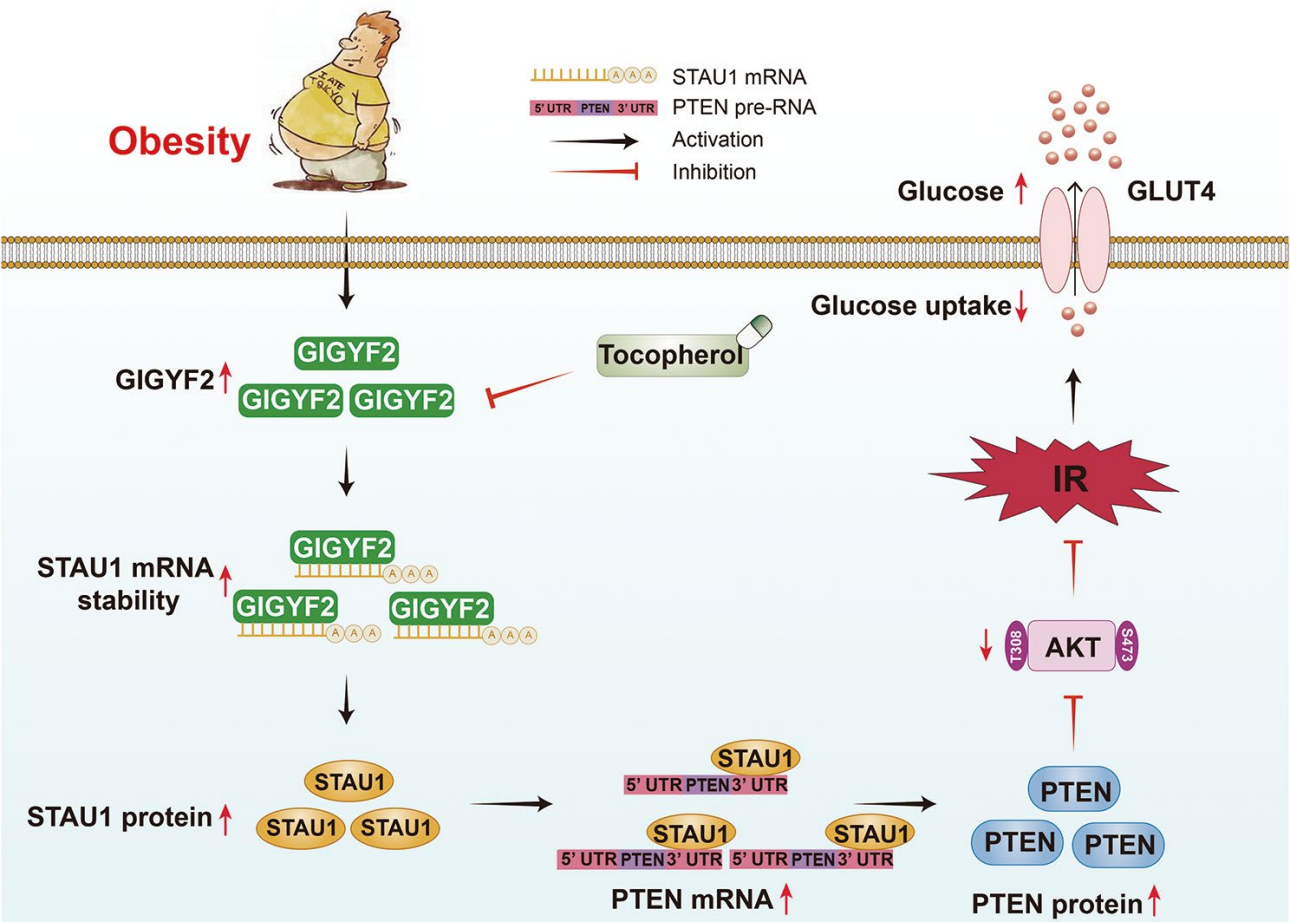

**Supplementary Information:**

The online version contains supplementary material available at 10.1186/s10020-024-00889-6.

## Introduction

Insulin resistance (IR) is a pathological condition in which insulin promotes reduced glucose uptake and utilization due to environmental and genetic variations, resulting in reduced sensitivity and responsiveness of the body to the physiological effects of insulin (Santoleri and Titchenell 2019). A bulk of study evidence demonstrates that IR is a common characteristic among several metabolic disorders, including metabolic syndrome, obesity, dyslipidemia, atherosclerosis, and diabetes (Yaribeygi et al. 2018). Obesity is widely recognized as a worldwide health concern with a constantly rising prevalence (Jaacks et al. 2019). In obese individuals, heavy traffic of lipids results in the release of excess triglycerides into the circulation as free fatty acids (FFAs). Importantly, the accumulation of these FFAs in non-adipose tissues, including the liver, also is implicated in the development of IR. Whereas, the underlying molecular mechanisms by which FFAs modulate hepatic IR have not been well elucidated.

Phosphoinositide 3-kinase, commonly known as PI3K, acts by phosphorylating the inositol ring of phosphatidylinositol at the 3-position to produce phosphatidylinositol (3,4,5)-trisphosphate (PIP3) with serine/threonine (Ser/Thr) activity. AKT, also known as protein kinase B (PKB), is a Ser/Thr kinase and a key player in the PI3K downstream signaling network (Zhang et al. 2019). It is widely accepted that the PI3K/AKT signaling pathway is essential for maintaining glucose homeostasis (Huang et al. 2018). Within the insulin-mediated signal transduction network, AKT undergoes phosphorylation by PI3K following the activation of the insulin receptor β-subunit (Beg et al. 2017). Notably, AKT activation stimulates various downstream molecules, including the translocation of glucose transporters from the cytoplasm to the plasma membrane, thereby enhancing glucose uptake (Beg et al. 2017). In a wide range of cells and tissues, substantial evidence has revealed that defects in the PI3K/AKT pathway or its downstream molecules strongly contribute to the pathogenesis of IR (Wu et al. 2018). In the context of obesity, impairment of the PI3K/AKT pathway provoked by obesity across multiple tissues contributes to IR and type 2 diabetes (T2D). Conversely, IR further impairs the PI3K/AKT pathway, creating a feedback loop that exacerbates both conditions (Huang et al. 2018). Whereas, the mechanisms by which obesity disrupts the PI3K/AKT signaling cascade still remain incompletely understood to date.

The Grb10-Interacting GYF Protein 2 (GIGYF2) gene is located on human chromosome 2q37, a region linked to familial Parkinson’s disease, and an intimate association between GIGYF2 mutations and Parkinson’s disease has been revealed (Ruiz-Martinez et al. 2015; Sutherland et al. 2009). GIGYF2 was initially identified as a Grb10 N-terminal interacting protein via the yeast two-hybrid screening (Giovannone et al. 2003). Recently, we found that GIGYF2 could function as an RNA-binding protein (RBP) to promote the activation of mTORC1-S6K1 signaling by recruiting mTORC1 to the lysosomal membrane, in turn leading to endothelial cell senescence, dysfunction, and vascular aging (Niu et al. 2023). Moreover, Grb10, as an adaptor protein, interacts with tyrosine-phosphorylated growth factor receptors such as insulin-like growth factor 1 receptor (IGF1R) and insulin receptor to serve as an endogenous negative regulator of IGF1R signaling (Dufresne and Smith 2005; Langlais et al. 2004). In the primary cultured embryonic fibroblasts from GIGYF2-deficient mice, reduced insulin-like growth factor 1 (IGF1)-stimulated IGF1R tyrosine phosphorylation was accompanied by increased activation of extracellular signal-regulated kinase 1/2 (ERK1/2) signaling (Giovannone et al. 2009). Likewise, a significant increase in GIGYF2 expression has been found to suppress IGF1R phosphorylation in a streptozotocin-induced diabetic mouse model, along with inactivation of downstream signaling of IGF1R, including AKT and ERK1/2 pathways (Blum et al. 2014). Furthermore, elevated GIGYF2 expression may facilitate the development of diabetes-associated cognitive impairment by negatively regulating the IGF1R signaling pathway. Mountains of evidence has demonstrated that the IGF1R, AKT, and ERK1/2 are strongly involved in the pathogenesis of IR (Dong et al. 2019; Fernández et al. 2001). These studies implicate that GIGYF2 may exert a unique function in orchestrating IR that contributes to the development of diabetes.

As a target organ for insulin action, the liver with abundant expression of GIGYF2 is crucial for maintaining glucose homeostasis in the human body (Molinaro et al. 2020). In obese mammals, the liver often exhibits impaired responsiveness to insulin, leading to hepatic insulin resistance and excessive conversion of glucose into fat, which in turn results in fatty liver disease and liver damage (Sakurai et al. 2021). Palmitic acid (PA), the most common circulating saturated FFAs, and high levels of PA exposure are extensively documented to trigger IR and T2D (Reynoso et al. 2003). Nevertheless, little is known about GIGYF2, and its potential link with IR in the liver has been barely documented. Based on these contexts, here we sought to elucidate the role of GIGYF2 and its underlying mechanism of action in modulating insulin resistance and diabetes development in the context of obesity.

## Research design and methods

### Materials

Reagents used in our experiment were acquired from various suppliers: rabbit antibodies against GIGYF2 (24790-1-AP, Proteintech Wuhan, China), STAU1 (14225-1-AP, Proteintech Wuhan, China), rabbit anti-total AKT (#4685s, CST, USA), phospho-AKT (Ser 473) (#4060s, CST, USA), phospho-AKT (Thr308) (#13038s, CST, USA); β-actin mouse monoclonal antibody (#4970s, CST, USA); mouse anti-PTEN antibodies (60300-1-IG, Proteintech Wuhan, China). Plasmids (PSPAX2, PMD2.G) were acquired from Vector Builder (China). Tocopherol (α-tocopherol) was from MACKLIN (D832569, MACKLIN, China). Cell counting Kit-8 assay was obtained from Beyotime (Cat No.C0037, Beyotime, China). The pmirGLO Dual-Luciferase expression vector was sourced from Promega (USA).

### Formulation of PA solution

PA was conjugated to BSA for cell treatment. Briefly, 0.1025 g of PA was added to 1 mL of 400 mM NaOH solution. The mixture was shaken and incubated in a 90 °C water bath to ensure complete saponification. The solution was then agitated until transparent and colorless. Subsequently, 7 mL of 10% BSA (fatty acid-free) solution was rapidly mixed with the PA solution. After vigorous shaking and stirring, the combined solution was filtered in a 55 °C water bath. The pH was adjusted to 7.2–7.4 using either sodium hydroxide or hydrochloric acid. The final solution contained 50 mM palmitate and was stored at -80 °C. For cell experiments, hepatocytes were exposed to varying concentrations of PA solution (0.1, 0.3, 0.5, 0.7 mM) prepared as described above. Control cells received only 10% of BSA treatment.

### Cell cultures

The human hepatoma cell line HepG2 and human embryonic kidney cell HEK-293T used in our study were purchased from ATCC (Manassas, USA). All cells were cultivated in Dulbecco’s Modified Eagle’s Medium (DMEM; Sigma, USA) at 37 °C with a humidity chamber containing 5% CO_2_ and 95% air. All cell culture media were supplemented with 10% fetal bovine serum (Sigma, USA) and 1% Penicillin-Streptomycin (Beyotime, China).

### Measurement of extracellular glucose

The Glucose Assay Kit (AKSU001M, Boxbio, China) instructions were used to determine the amount of glucose present in the conditioned medium. To make the working mixes, combine reagents I and II in equal volumes. 100 µL of standards or samples should be added to 900 µL of working mixture, and then incubated for 15 min at 37 °C. Real-time UV–visible spectrophotometer readings at 505 nm were used to determine the absorbance value. The standard curve was generated using the absorbance values for glucose concentrations of 0.4, 0.3, 0.2, 0.1, 0.05, and 0.025 mg/mL.

### Cell glucose uptake assay

Cells were stained with the fluorescent dye 2-NBDG (B6035, APExBIO, USA) to assess glucose uptake. After various treatments, the cells were washed with PBS and centrifuged at 800 rpm for 5 min, then incubated with 200 µM glucose fluorescent analogue 2-NBDG in a glucose-free DMEM medium at 37 °C for 80 min, kept away from light. After incubation, cells were washed with a basal glucose-free DMEM medium and centrifuged at 800 rpm for 5 min. The fluorescence signal was monitored using a fluorescence microscope with an FITC filter and quantified by NIH Image J software (NIH, Bethesda, MD). The fluorescence signals are also monitored using a flow cytometer with a FITC channel.

### Western blot analysis

Cells were lysed using RIPA buffer to prepare protein extracts. Protein concentration was determined using the Pierce BCA Protein Assay kit. Subsequently, 20 µg of denatured protein extracts were separated by electrophoresis on a 10-12.5% SDS-polyacrylamide gel and transferred to nitrocellulose membranes. The membranes were blocked with 5% skimmed milk and then incubated with primary antibodies at room temperature for 2 h or 4 °C overnight. Following primary antibody incubation, membranes were washed and incubated with corresponding secondary antibodies for 1.5 h at room temperature. Protein bands were visualized using a chemiluminescence imaging system (Tanon, China) and quantified by densitometry using ImageJ software. β-ACTIN served as a loading control.

### Generation of lentivirus and expression vectors for genes overexpression and knockdown

HEK-293T cells were co-transfected with the packaging plasmid (psPAX2), envelope plasmid (pMD2.G), and the PLKO.1-TRC plasmid with targeted shRNA sequences for knockdown or PLJMI plasmid with STAU1 gene sequences for overexpression using Lipo6000™ Transfection Reagent to produce the corresponding lentivirus. After culturing the transfected HEK-293T cells for 24 h and 48 h, the supernatant was removed and centrifuged for 5 min at 1000 rpm to extract virus particles. Subsequently, lentivirus-infected cells were screened for cells expressing the relevant antibiotic resistance gene in a growth medium supplemented with 2 µg/mL puromycin.

For overexpression of GIGYF2, the GIGYF2 gene underwent amplification from the template plasmid PX459-HA-GIGYF2 via PCR, employing the forward primer: 5′-CGCTAGCGCTACCGGCACCATGGCCTACCCATATGATG − 3′, and the reverse primer sequence:

5′-TCGAGGTCGAGAATTTCAGTAGTCATCCAACGTCTCGA − 3’. To construct the STAU1 overexpression vector, the STAU1 gene was amplified from genomic DNA extracted from human umbilical vein endothelial cells (HUVECs) using PCR. The forward primer sequence was 5′-CGCTAGCGCTACCGGCACCATGAAACTTGGAAAAAAACCA-3′, and the reverse primer sequence was 5′-CCGAGGTCGAGAATTTCAGCACCTCCCACACACAG-3′. To interfere with GIGYF2 expression, short hairpin RNA (shRNA) oligos of GIGFY2 or STAU1 or PTEN targeting GIGYF2 were cloned into pLKO.1. The boldface sequences below represent the targeting sequences for hGIGYF2-shRNA, hSTAU1-shRNA, and hPTEN-shRNA (only the sense strand is shown):

pLKO.1-hGIGYF2-F:

5′CCGG**CACAGTACACTCCATTCAGTA**CTCGAGTACTGAATGGAGTGTACTGTGTTTTTG-3′.

pLKO.1-hSTAU1-F:

5′CCGG**GCCTGCAGTTGAACGAGTAAA**CTCGAGTTTACTCGTTCAACTGCAGGCTTTTTG-3’;

pLKO.1-hPTEN-F:

5’CCGG**CCACAAATGAAGGGATATAAA**CTCGAGTTTATATCCCTTCATTTGTGGTTTTTG-3’.

### Quantitative real-time PCR

Total RNA was isolated from cells using TriQuick Reagent according to the manufacturer’s instructions. To evaluate mRNA expression levels, two-step quantitative PCR (qPCR) was performed. GAPDH was used as a reference gene for normalization. The specific oligonucleotide primer sequences are listed in Table 1.

**Table 1:** List of primers used for qRT-PCR

Target gene	Forward	Reverse
hGIGYF2 hSTAU1 hGAPDH hPTEN	ATCTTCCTCTGGACACCACG TGCACTTAAACGGAACTTGCC TGCACCACCAACTGCTTAGC TGAGTTCCCTCAGCCGTTACCT	GTCGCCGAAGAATTTCCTCC AATCGGATTGATCCCCTGGC GGCATGGACTGTGGTCATGAG GAGGTTTCCTCTGGTCCTGGTA

h: human

### mRNA stability assay

After transfection, cells were exposed to 10 µg/mL of Actinomycin D and collected in TRIzol at 0, 2, 4, and 6 h to determine the rates of mRNA decay. The percentage of the target gene’s remaining mRNA was then determined using qRT-PCR tests. GAPDH was used to normalize the mRNA levels.

### RNA immunoprecipitation

After a quick wash in ice-cold PBS, the cells were lysed on ice for 30 min using a lysis buffer supplemented with protease inhibitors (GLPBIO) containing 40 mM HEPES (pH 7.5), 1 mM EDTA, 10 mM pyrophosphate, 120 mM NaCl, 1 mM EDTA, 10 mM glycerol phosphate, and 0.3% CHAPS. The cells were then scraped, and the supernatant was transferred to a new 1.5 mL microcentrifuge tube after the cells were centrifuged at 10,000 ×g for 15 min at 4 °C to remove debris. For the RNA input control, 490 µL of TRIzol was combined with 1% of the supernatant sample. Protein A/G agarose beads (Santa Cruz) were used to pre-cleared the lysates, and then they were treated with primary antibodies for 2 h at 4 °C. After that, they were incubated with 20 µL A/G beads for an entire night at 4 °C with gentle rotation. The immunoprecipitate was obtained by centrifugation at 2500 rpm at 4 °C for 5 min and washed 3–4 times with washing buffer containing 50 mM HEPES (pH 7.5), 40 mM NaCl, and 2 mM EDTA. The beads with the immunoprecipitated samples were resuspended in 500 µL TRIzol and processed for qRT-PCR analysis.

### Luciferase activity assay

The Dual-Luciferase Assay Kit (Promega, USA) was used to perform the luciferase activity assay following the guidelines provided by the manufacturer. The wild-type and mutant STAU1 DNA fragments (Table 2) were cloned using the pmirGLO Dual-Luciferase Vector (Promega, USA), and then co-transfected with the pLJM1-STAU1 plasmid using the Lipo6000™ transfection reagent into 70% confluent HEK-293T cells. After 48 h of transfection, cells were lysed with the passive buffer and centrifuged at 12,000 rpm for 10 min at 4 °C. The supernatant was then gathered, and the luciferase activity was measured on a BioTek microplate reader.

**Table 2:** List of DNA fragments of wild-type and mutant sequences

Position	Target	DNA fragments
chr7:87863909–87,863,935, chr7	hSTAU1-PTEN-WT-F1: hSTAU1- PTEN-WT-R1: hSTAU1- PTEN-MUT-F1: hSTAU1- PTEN-MUT-R1:	5’-GCTGCACAC**AAAAAAAA**GACATTTGA-3’ 5’-CTAGTCAAATGTC**TTTTTTTT**GTGTGCAGCAGCT-3’ 5’-GCTGCACAC**AGACATGA**GACATTTGA-3’ 5’-CTAGTCAAATGTC**TCATGTCT**GTGTGCAGCAGCT-3’
chr7: 87,864,085–87,864,111, chr7	hSTAU1- PTEN-WT-F2: hSTAU1- PTEN-WT-R2: hSTAU1- PTEN-MUT-F2: hSTAU1- PTEN-MUT-R2:	5’-TCACTTCTT**AAAAAAAA**TCATCATAT-3’ 5’-CTAGATATGATGA**TTTTTTTT**AAGAAGTGAAGCT-3’ 5’-TCACTTCTT**CACAGCAG**TCATCATAT-3’ 5’-CTAGATATGATGA**CTGCTGTG**AAGAAGTGAAGCT-3’
chr7: 87,863,784–87,863,808, chr7	hSTAU1- PTEN-WT-F3: hSTAU1- PTEN-WT-R3: hSTAU1- PTEN-MUT-F3: hSTAU1- PTEN-MUT-R3:	5’-TTGCACATT**TTTTAA**ATGTCATTA-3’ 5’-CTAGTAATGACAT**TTAAAA**AATGTGCAAAGCT-3’ 5’-TTGCACATT**CACTAG**ATGTCATTA-3’ 5’-CTAGTAATGACAT**CTAGTG**AATGTGCAAAGCT-3’
chr7: 87,864,128–87,864,152, chr7	hSTAU1- PTEN-WT-F4: hSTAU1- PTEN-WT-R4: hSTAU1- PTEN-MUT-F4: hSTAU1- PTEN-MUT-R4:	5’-TGCATACGA**TTTTAA**GCGGAGTAC-3’ 5’-CTAGGTACTCCGC**TTAAAA**TCGTATGCAAGCT-3’ 5’-TGCATACGA**TCGTCA**GCGGAGTAC-3’ 5’-CTAGGTACTCCGC**TGACGATC**GTATGCAAGCT-3’
chr7: 87,865,188–87,865,212, chr7	hSTAU1- PTEN-WT-F5: hSTAU1- PTEN-WT-R5: hSTAU1- PTEN-MUT-F5: hSTAU1- PTEN-MUT-R5:	5’-AGGCATCAC**TTTTAA**GAAAGCTTA-3’ 5’-CTAGTAAGCTTTC**TTAAAA**GTGATGCCTAGCT-3’ 5’-AGGCATCAC**TCGTCA**GAAAGCTTA-3’ 5’-CTAGTAAGCTTTC**TGACGAGT**GATGCCTAGCT-3’

The bases in bold are the predicted binding sites of STAU1 protein and PTEN RNA

### Animal

The 4-week-old C57BL/6J male mice were purchased from Cyagen Bioscience (Suzhou, China) and all had free access to food and water and were maintained in a room controlled by temperature (22 ℃ to 25 ℃), humidity (50%) and light (12 h light/dark cycle). After 2 weeks, the mice were randomly divided into four groups (*n* = 6 per group): the normal control group in which mice took a normal diet (NCD; 10% kcal of fat, Research Diets: D12450H) for 12 weeks; the HFD group in which mice took a high-fat diet (HFD; 60% kcal of fat; Research diet: D12492) for 12 weeks; the HFD + lentivirus-knockdown GIGYF2 group in which mice took a high-fat diet for 12 weeks, starting from week 8, the mice received knocking down GIGYF2 lentivirus (1 × 10^9^ pfu/100 µL) via tail vein injection 2–3 times per week for 4 weeks; the HFD + tocopherol group in which HFD was replaced by tocopherol supplemented-HFD (800 mg/kg diet) (Kiyose et al. 2021) (Peluzio et al. 2003) (Desrumaux et al. 2013) (Kaliappan et al. 2013) after 8 weeks of HFD feeding. During the experiment, the body weight of mice was measured twice a week. Glucose tolerance test (GTT) and insulin tolerance test (ITT) were performed to assess the insulin resistance of mice. Following these tests, all mice were euthanized, and liver weight was recorded. Liver tissues were then collected and stored at -80 °C for subsequent analysis. All animal experiments were conducted with the approval of the Animal Ethics Review Committee of Northwestern University (NWU-AWC-20221105 M) and carried out in accordance with the guidelines from EU Directive 2010/63/EU.

### Statistics

All data were obtained from three independent experiments. Comparisons of gene expression between groups were analyzed using unpaired t-tests or one-way ANOVA with Bonferroni post test to obtain p-values. Statistical analyses were performed using GraphPad Prism 8.0. Non-significant differences were indicated as “n.s.” and *p* < 0.05 was considered statistically significant.

## Results

### GIGYF2 is significantly elevated in PA-induced hepatocytes IR

**Fig. 1:**
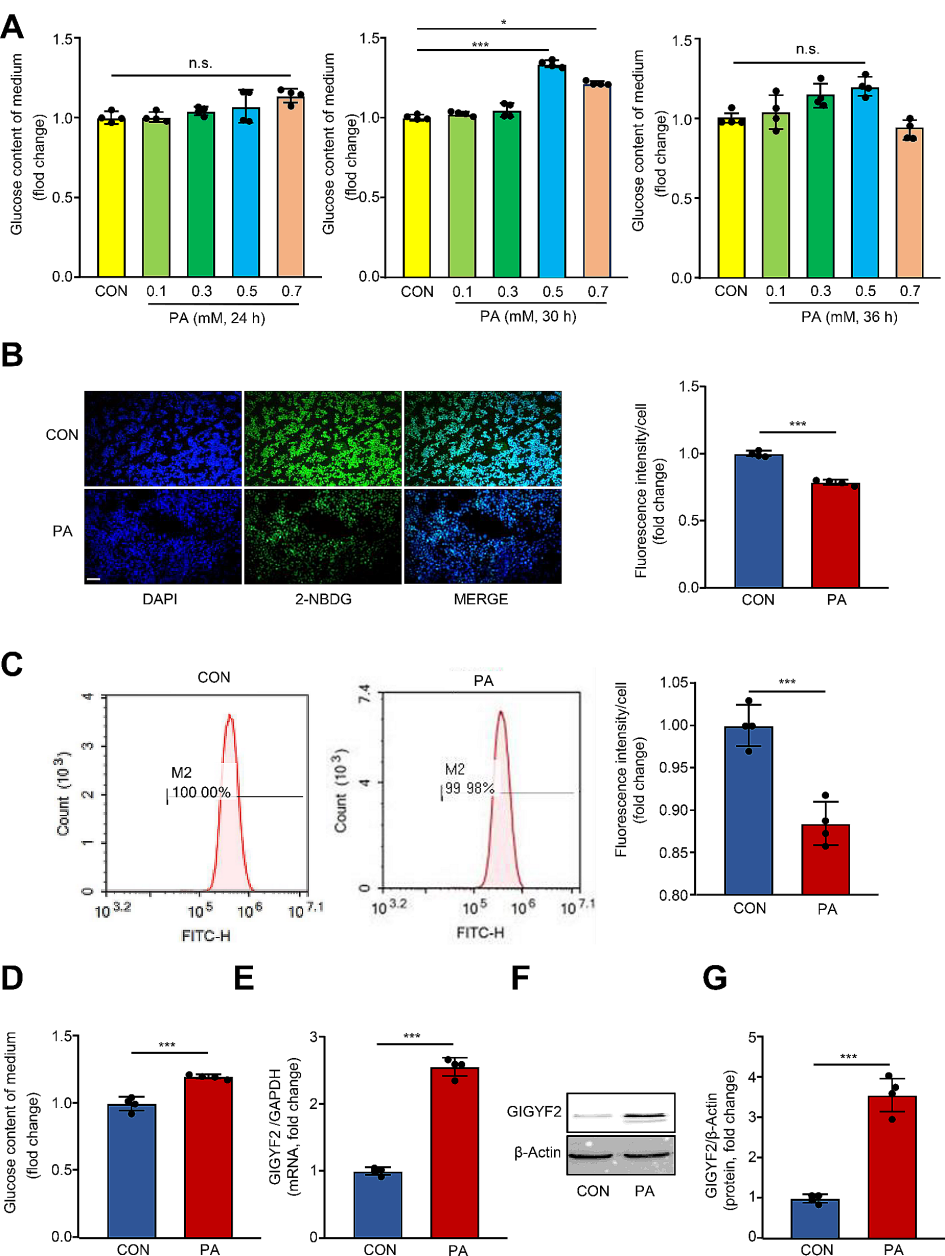
GIGYF2 is markedly up-regulated in PA-induced IR in hepatocytes. (**A**) HepG2 cells were treated with 10% BSA (CON) or various concentrations of PA (0.1 mM, 0.3 mM, 0.5 mM, and 0.7 mM). D-Glucose content assay kit detected the glucose content of the medium at 24, 30, and 36 h time points. (**B**) Representative images show the fluorescence intensity of 2-NBDG uptake in HepG2 cells cultured under a 30 h incubation with 0.5 mM PA. The bar chart on the right shows the quantization of the intensity signaling. (**C**) The 2-NBDG uptake rate of the control group and 0.5 mM PA group was detected by flow cytometric analysis. (**D**) The glucose content of the medium of CON and PA groups. (**E**) The mRNA and (**F**-**G**) protein levels of the GIGYF2 in CON and PA treatment groups. Scale = 100 μm. n.s.: not significant, *n* = 4, *p < 0.05, ***p < 0.001

A high dose of PA exposure suppresses the insulin signaling pathway in various cells and tissues, resulting in glucose intolerance and impaired insulin sensitivity (Palomer et al. 2018). Here, we treated HepG2 cells with different PA dosages (0.1 mM, 0.3 mM, 0.5 mM, and 0.7 mM) and time durations (24 h, 30 h, and 36 h) to establish the PA-induced IR model in hepatocytes. As shown in Fig. 1A, the glucose content of the conditioned medium reached a maximum peak upon 0.5 mM PA exposure for 30 h. Meanwhile, the 2-NBDG fluorescence staining confirmed that this does of PA treatment condition dramatically reduced intracellular glucose uptake (Fig. 1B-C). Additionally, compared to untreated cells, PA treatment (0.5 mM, 30 h) induced a remarkable increase in glucose content in the culture medium (Fig. 1D), demonstrating that treatment with 0.5 µM PA for 30 h tends to develop IR in hepatocyte cells. To investigate whether GIGYF2 is implicated in PA-induced hepatic IR, we analyzed the expression of GIGYF2 under this condition. Strikingly, qRT-PCR and western blot analysis assays showed that PA-induced IR was accompanied by dramatic upregulation of GIGYF2 at mRNA (Fig. 1E) and protein (Fig. 1F-G) expression levels in HepG2 cells.

### Silencing GIGYF2 ameliorates PA-induced IR in hepatocytes

**Fig. 2:**
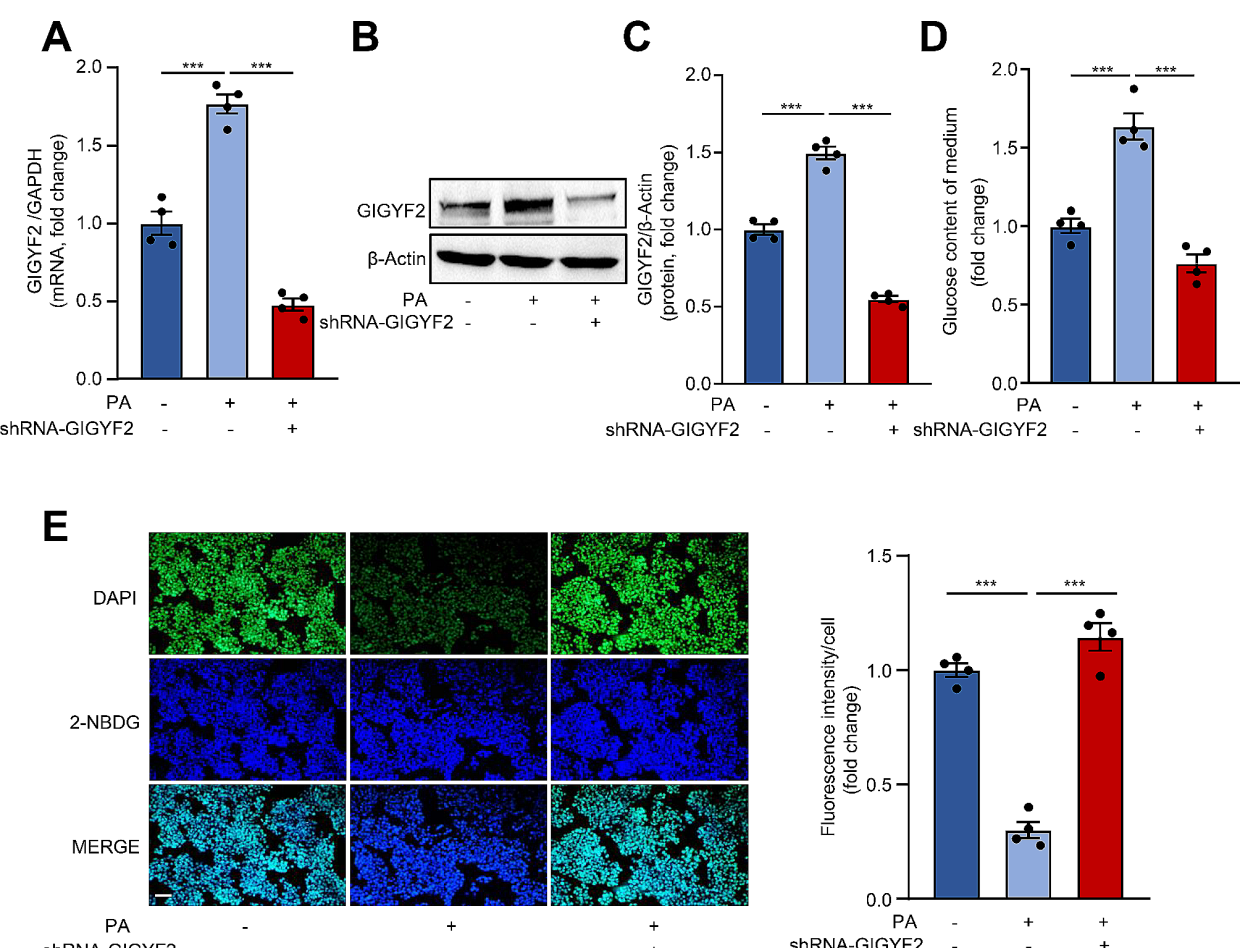
Silencing GIGYF2 ameliorates PA-induced IR in hepatocytes. HepG2 cells were transfected for 8 h with negative control and knockdown lentivirus shRNA-GIGYF2 for silencing GIGYF2 and then treated with PA for 30 h. (**A**) qPCR analysis of the GIGYF2 mRNA levels. (**B**-**C**) Western blotting analysis of GIGYF2 protein level. (**D**) Measurement of glucose content of the medium. (**E**) Fluorescent staining analysis of 2-NBDG uptake. The bar graph on the right shows the quantification of fluorescent intensity signaling. Scale = 100 μm. *n* = 4, **p < 0.01, ***p < 0.001

To interrogate whether GIGYF2 mediates PA-induced IR in hepatocytes, we used lentivirus-mediated shRNA to silence GIGYF2. qRT-PCR and western blot verified the knockdown efficiency of GIGYF2 at mRNA (Fig. 2A) and protein (Fig. 2B-C) levels, respectively. Subsequently, the glucose content in the conditioned medium and intracellular glucose uptake were then determined. As shown in Fig. 2D, silencing GIGYF2 is prominent in preventing PA-induced increase in glucose content in the conditioned medium. Furthermore, silencing GIGYF2 markedly enhanced the intracellular glucose uptake as compared to the PA-treated group (Fig. 2E). These results demonstrate that GIGYF2 is implicated in regulating PA-induced IR in hepatocytes.

### Overexpression of GIGYF2 promotes IR, PTEN upregulation and PI3K/AKT inactivation in HepG2 cells

**Fig. 3:**
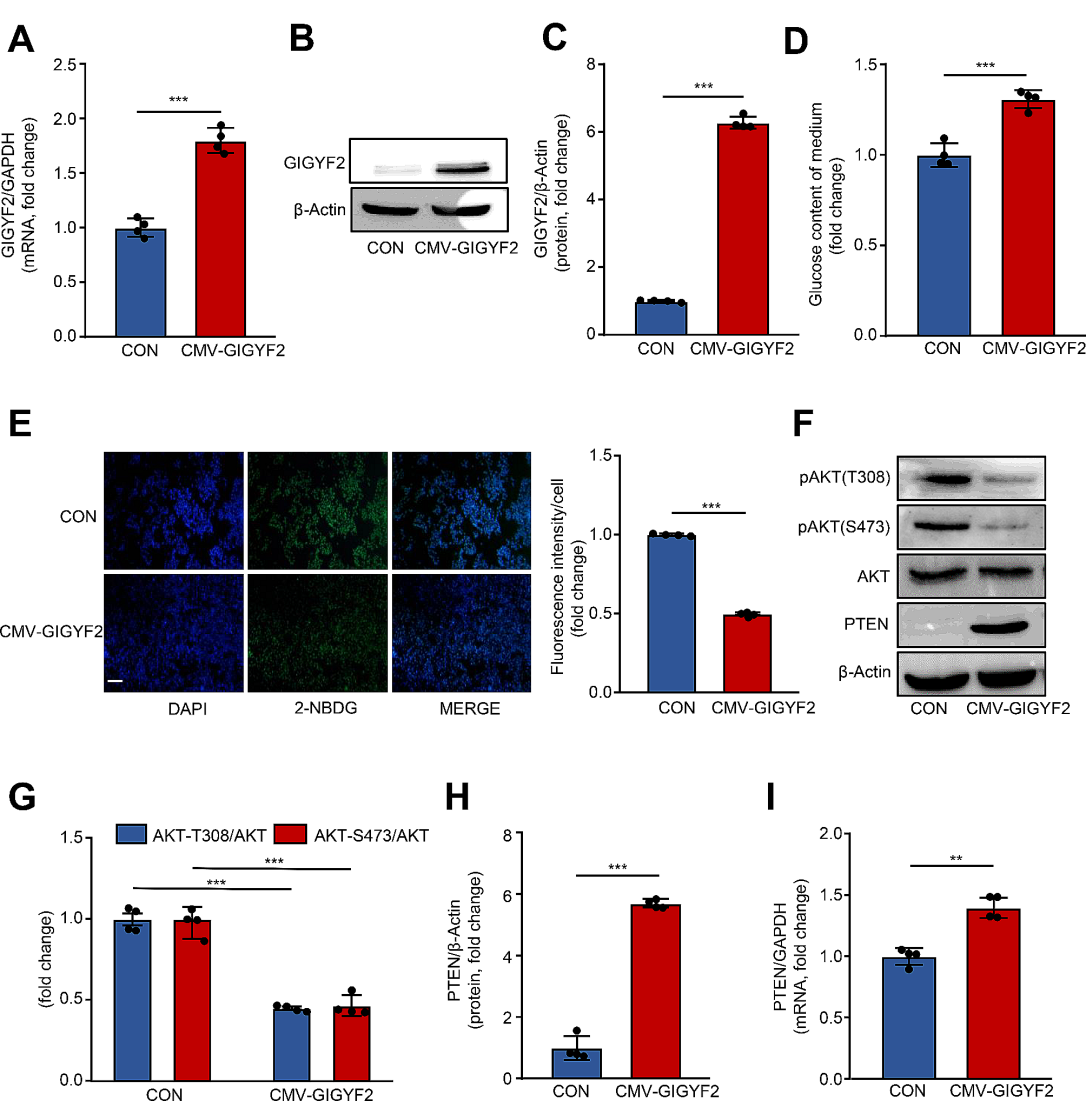
Overexpression of GIGYF2 promotes IR, PTEN elevation, and PI3K/AKT inactivation in hepatocytes. HepG2 cells were transduced with overexpressed CMV empty lentivirus as control (CON) or CMV-GIGYF2. The cells were subjected to (**A**) qPCR analysis of the GIGYF2 mRNA levels and (**B**-**C**) western blotting analysis of GIGYF2 protein levels. (**D**) Measurement of glucose content of the medium. (**E**) The fluorescent staining analysis of 2-NBDG uptake. (**F**) Western blotting analysis of the expression levels of PTEN, AKT, AKT-Thr308, and AKT-Ser473 in CON and GIGYF2 groups. (**G**) Quantification of relative levels of AKT-Thr308 and AKT-Ser473 proteins normalized to AKT. (**H**) Represents quantification of the relative levels of PTEN protein normalized to β-actin. (**I**) qRT-PCR analysis of the expression of the mRNA level of PTEN. Scale = 100 μm. *n* = 4, **p < 0.01, ***p < 0.001

Next, by using the lentivirus-mediated ectopic GIGYF2 overexpression, we investigated the impact of GIGYF2 per se on glucose uptake and insulin sensitivity without PA stimulation in HepG2 cells. qRT-PCR (Fig. 3A) and western blot (Fig. 3B-C) confirmed the overexpression of GIGYF2 in HepG2 cells. As expected, the overexpression of GIGYF2 significantly enhances the glucose content of the conditioned medium (Fig. 3D) and impairs intracellular the cellular glucose uptake (Fig. 3E), respectively. These results provide evidence for a definite role of GIGYF2 in promoting IR in hepatocytes. The impairment of the PI3K/AKT pathway has been widely recognized to promote IR, which prompted us to investigate whether GIGYF2 mediates IR via the PI3K/AKT signaling (Ramasubbu and Devi Rajeswari 2022). Indeed, overexpressing GIGYF2 in HepG2 cells dramatically reduces the AKT phosphorylation levels at Thr308 and Ser473 as confirmed by western blot (Fig. 3F-G). More importantly, we also observed the PA significantly suppressed the PI3K/AKT signaling, while this could be blocked by the knockdown of GIGYF2 (Supplementary Fig. 1A-B). Additionally, we also examined the expression of the PTEN that negatively mediated the PI3K/AKT pathway. Intriguingly, overexpressing GIGYF2 remarkably elevated the protein (Fig. 3F, H) and mRNA levels (Fig. 3I) of PTEN. Moreover, PA exposure markedly downregulated the PTEN expression, which was also prevented by silencing GIGYF2 (Supplementary Fig. 1A, C). These results prompt us to speculate that GIGYF2 may promote IR through PTEN-mediated inactivation of the PI3K/AKT signaling pathway in hepatocytes.

### GIGYF2 promotes IR through PTEN-mediated inactivation of the PI3K/AKT pathway

**Fig. 4:**
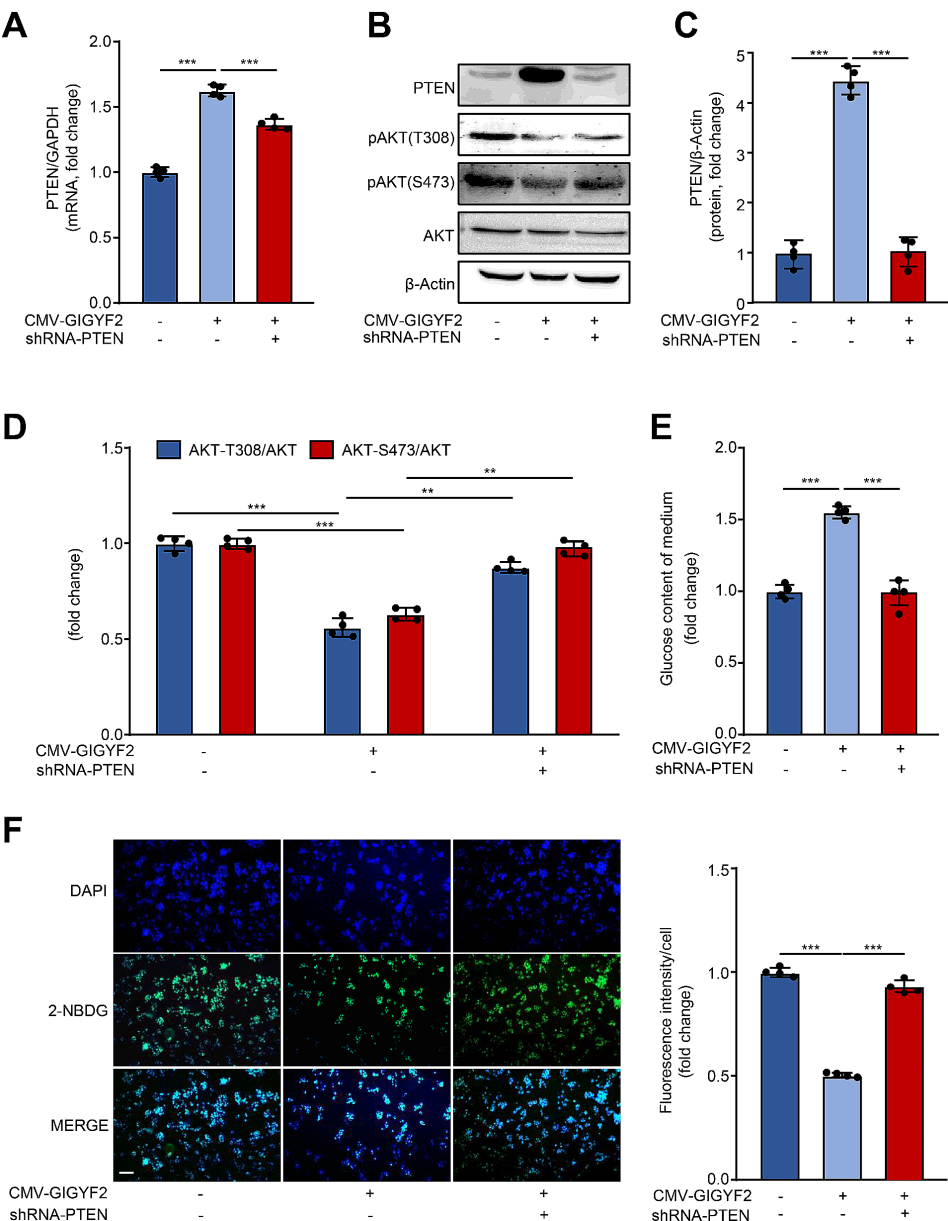
GIGYF2 induces IR through PTEN-mediated inactivation of the PI3K/AKT pathway in hepatocytes. HepG2 cells were transduced lentivirus CMV-empty as control or CMV-GIGYF2. After 24 h, cells were transduced with lentivirus-mediated PTEN shRNA for silencing PTEN. 48 h post-transduction, the cells were subjected to (**A**) qPCR analysis of the mRNA levels of PTEN. (**B**) Western blotting analysis of the protein levels of PTEN and AKT phosphorylation. (**C**) Quantification of the PTEN signals in (**B**). (**D**) Quantification of the AKT phosphorylation signals in (**B**). (**E**) The glucose content of the medium. (**F**) Representative images of the fluorescence signaling of 2-NBDG for evaluating glucose uptake. Scale bar = 100 μm. *n* = 4, **p < 0.01, ***p < 0.001

To verify the above speculation, we employed PTEN depletion via the lentivirus-mediated PTEN shRNA as confirmed by qPCR (Fig. 4A) and western blotting (Fig. 4B-C) in HepG2 cells upon GIGYF2 overexpression. In line with expectations, we found that the depletion of PTEN significantly reversed the inhibition of AKT phosphorylation at Thr308 and Ser473 induced by GIGYF2 overexpression (Fig. 4B, D). Meanwhile, the increased glucose content of the supernatant (Fig. 4E) and reduced glucose uptake (Fig. 4F) induced by GIGYF2 overexpression were reversed by depleting PTEN. These results reveal that GIGYF2 promotes IR through PTEN-mediated inactivation of the PI3K/AKT pathway in hepatocytes.

### GIGYF2 promotes PTEN elevation, PI3K/AKT inactivation and IR through STAU1

**Fig. 5:**
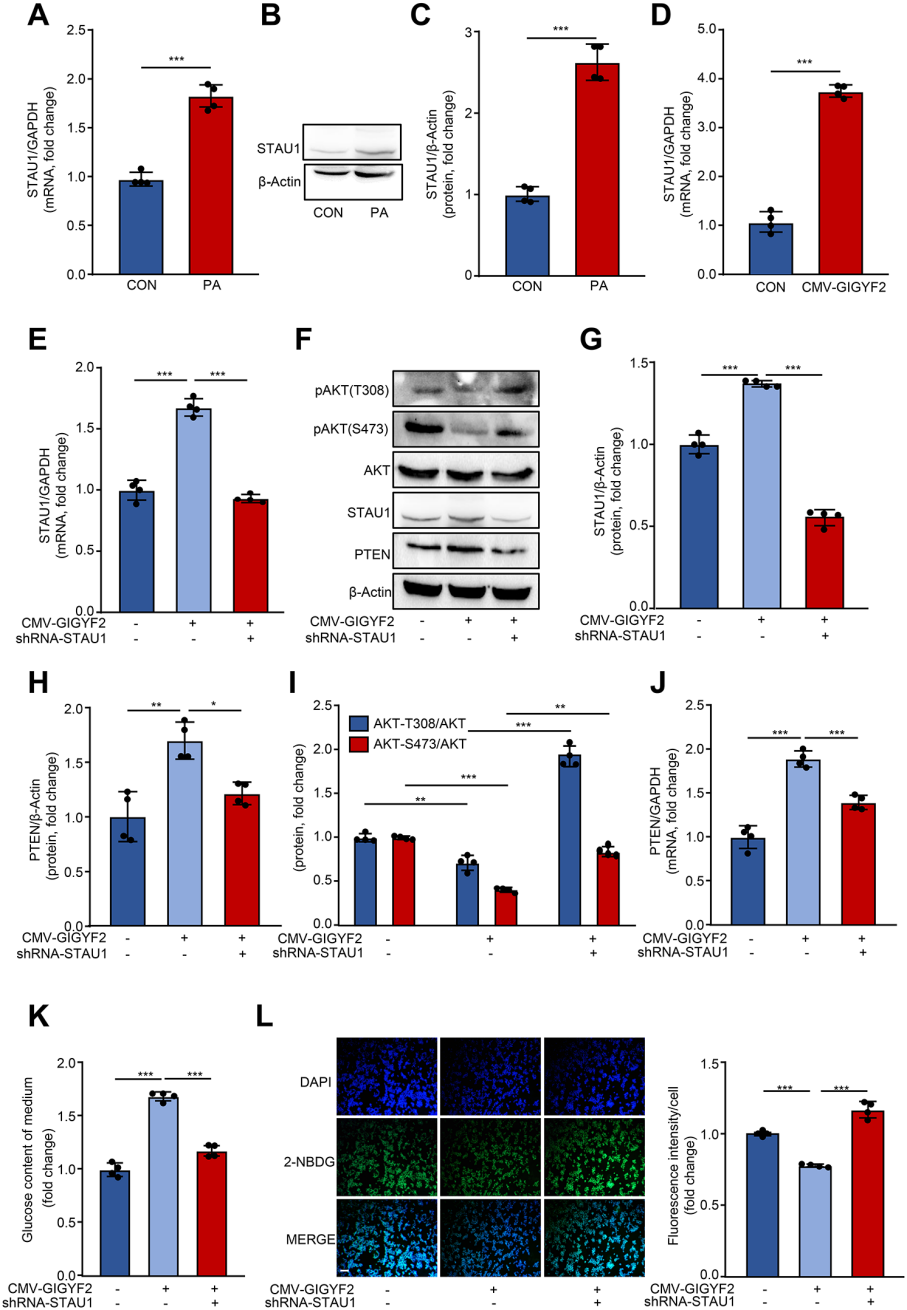
STAU1 deficiency prevents GIGYF2-induced PTEN elevation, PI3K/AKT pathway inactivation, and IR in HepG2 cells. (**A**) mRNA levels and (**B**) protein levels of STAU1 in control (CON) and PA groups. (**C**) Represents quantification of the relative levels of STAU1 protein normalized to β-actin. (**D**) HepG2 cells were transduced for two days with overexpressed CMV empty lentivirus as CON or CMV-GIGYF2, and the cells were subjected to qPCR analysis of the STAU1 mRNA levels. HepG2 cells were transduced with overexpression lentivirus CMV-empty as control or CMV-GIGYF2. 24 h post-transduction, cells were transduced with lentivirus-mediated STAU1 shRNA for silencing STAU1. 48 h post-transduction, the cells were subjected to (**E**) qPCR analysis of the mRNA levels of STAU1. (**F**) Western blotting analysis of the protein levels of GIGYF2, PTEN, STAU1, and AKT phosphorylation. (**G**) Quantification of the STAU1 signals in (**F**). (**H**) Quantification of the PTEN signals in (**F**). (**I**) Quantification of the AKT phosphorylation in (**F**). (**J**) qRT-PCR analysis of the expression of the mRNA level of PTEN. (**K**) The glucose content of the medium. (**L**) Representative images of the fluorescence signaling of 2-NBDG for evaluating glucose uptake. Scale bar = 100 μm. *n* = 4, *p < 0.05, **p < 0.01, ***p < 0.001

Next, we aimed to elucidate the precise molecular mechanism by which GIGYF2-mediated PTEN upregulation promotes PI3K/AKT pathway inactivation and IR in hepatocytes. Our previous transcriptional profiling analysis revealed that STAU1 was markedly downregulated in GIGYF2-depleted vascular endothelial cells (Niu et al. 2023). Intriguingly, here in HepG2 cells as exposure to PA with IR, the mRNA and protein expression levels of STAU1 were significantly upregulated (Fig. 5A-C). Moreover, in HepG2 cells without PA, overexpressing GIGYF2 per se also dramatically promoted the elevation of STAU1 mRNA expression levels (Fig. 5D), suggesting that STAU1 may positively orchestrate GIGYF2-induced IR. Next, we proceed to investigate whether GIGYF2 promotes PTEN expression elevation, PI3K/AKT signaling inactivation, and IR via upregulating STAU1. For this purpose, we carried out the lentivirus-mediated STAU1 deletion in HepG2 cells with GIGYF2 overexpression, as verified by qRT-PCR and western blot (Fig. 5E-G). We found that the upregulation of PTEN and inactivation of PI3K/AKT signaling caused by GIGYF2 were attenuated by STAU1 deficiency (Fig. 5F, H-J). Furthermore, silencing STAU1 also protected the HepG2 cells from GIGYF2-induced IR, e.g., decreased the glucose content of the culture medium and enhanced the intracellular glucose uptake (Fig. 5K-L). Consistently, in HepG2 cells without PA, overexpressing STAU1 per se (Supplementary Fig. 2A-C) also remarkably enhanced the glucose content in the conditioned medium (Supplementary Fig. 2D) and impaired intracellular glucose uptake (Supplementary Fig. 2E). Meanwhile, overexpressing STAU1 significantly boosted PTEN expression and suppressed AKT phosphorylation (Supplementary Fig. 2F-H). Next, we depleted PTEN in STAU1-overexpressed HepG2 cells (Supplementary Fig. 3A-C), showing that PTEN deficiency markedly prevented the inactivation of AKT evoked by STAU1 overexpression (Supplementary Fig. 3B, D). Importantly, depleting PTEN remarkably restored the STAU1-induced increase in glucose content of the conditioned medium and decrease in glucose uptake (Supplementary Fig. 3E-F). These results suggest that GIGYF2 functions as a positive regulator of STAU1, which in turn upregulates PTEN expression, ultimately leading to PI3K/AKT pathway inactivation and hepatic insulin resistance.

### STAU1 as an RNA-binding protein (RBP) enhances PTEN mRNA stability via binding to its 3’UTR

**Fig. 6:**
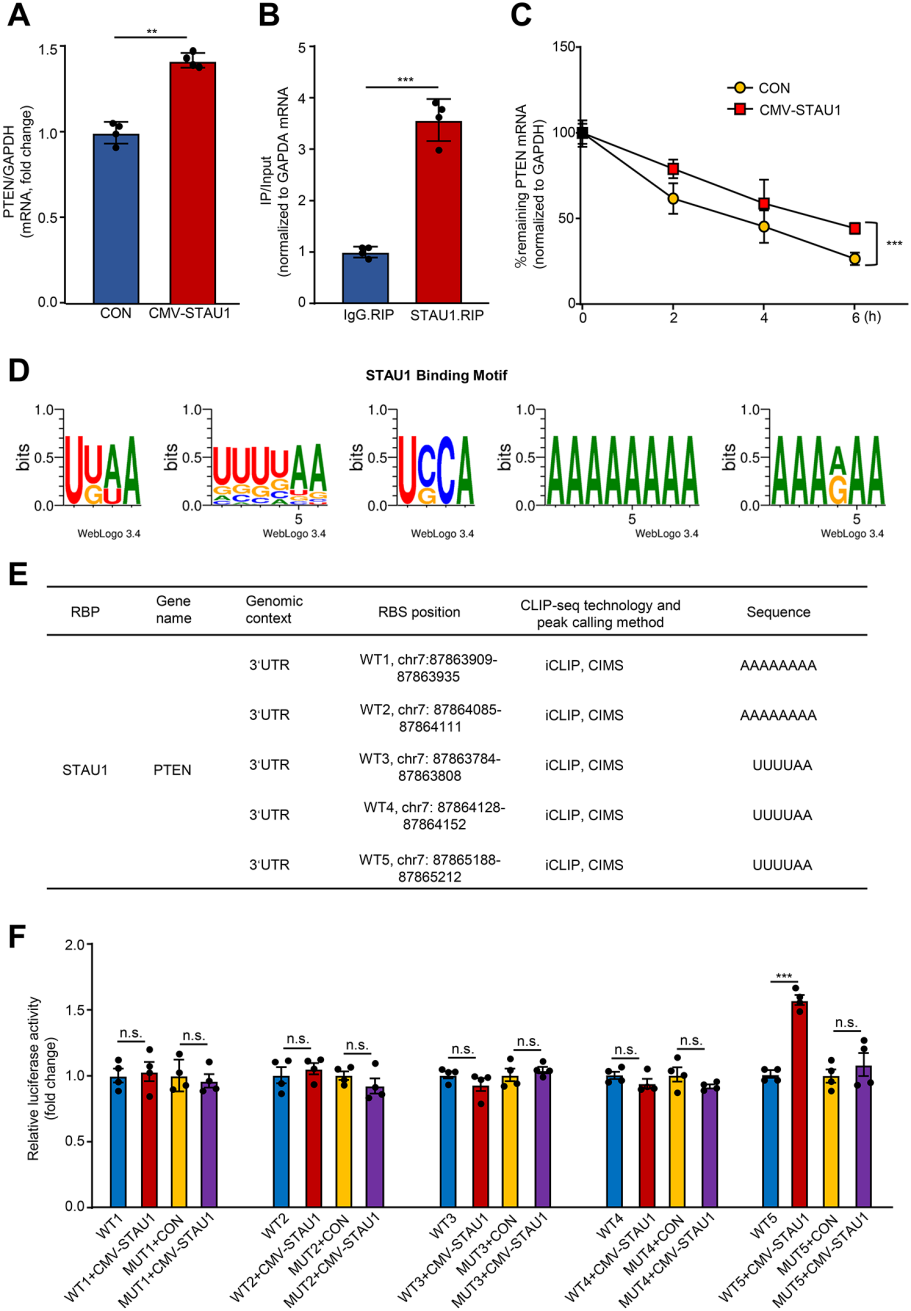
STAU1 as an RNA-binding protein enhances PTEN mRNA stability via the 3’UTR. HepG2 cells were transduced with overexpressed lentivirus of CMV-empty as control (CON) or CMV-STAU1 (STAU1) for two days. (**A**) The mRNA expression levels of PTEN in CON and STAU1 groups. (**B**) RIP assays were performed with control immunoglobulin G (IgG) and rabbit polyclonal antibodies directed against STAU1. The precipitated PTEN mRNA was analyzed by qRT-PCR. (**C**) HepG2 cells were transduced with CMV-empty as control (CON) or CMV-STAU1 (STAU1), and subsequently cells were treated with 10 µg/mL Actinomycin D for 0, 2, 4, and 6 h, followed by qRT-PCR to assess the percentage of remaining mRNA of STAU1 and GAPDH (reference gene). (**D**) POSTAR3 shows the potential binding motifs of PTEN mRNA with STAU1. (**E**) Binding regions (WT1, WT2, WT3, WT4 and WT5) of STAU1 and PTEN mRNA predicted by POSTAR3 database. (**F**) Luciferase reporting analysis of wild-type (WT1-5) and mutated (MUT1-5) STAU1 binding to PTEN RNA regions. n.s.: not significant. *n* = 4, **p < 0.01, ***p < 0.001

Curiously, we are wondering how STAU1 contributes to PTEN expression elevation. Given the fact that STAU1 is an RBP that can bind to gene RNA to regulate mRNA stability (Almasi et al. 2021), and our data indicate that overexpressing STAU1 markedly upregulates PTEN mRNA expression level in HepG2 cells (Fig. 6A), we thus propose that STAU1 as an RBP binding to PTEN mRNA enhances the PTEN mRNA stability. Indeed, RNA immunoprecipitation (RIP) confirmed the binding between STAU1 protein and PTEN mRNA (Fig. 6B). Furthermore, mRNA stability assays showed that hyperactive STAU1 expression enhances the PTEN mRNA stability (Fig. 6C). Notably, there are five potential binding motifs between STAU1 and the 3’UTR region of PTEN found in the POSTAR3 database (Fig. 6D). Accordingly, we constructed five corresponding wild-type (WT1-5) and mutated (MUT1-5) STAU1-PTEN binding sequences with different binding sites to examine the binding region of PTEN binding to STAU1 protein (Fig. 6E). Luciferase reporter assay demonstrated that the WT5 region (chr7: 87865188–87865212) but not WT1 region (chr7:87863909–87863935), WT2 region (chr7:87864085–87864111), WT3 region (chr7:87863784–87863808), and WT4 region (chr7:87864128–87864152) of PTEN 3’UTR could bind to STAU1 protein (Fig. 6F). These results suggest that STAU1 enhances the PTEN mRNA stability via binding to the 3’UTR of PTEN mRNA, resulting in elevation of PTEN expression.

### Phytopharmaceutical intervention of GIGYF2 by tocopherol attenuates PA-induced IR in hepatocytes

**Fig. 7:**
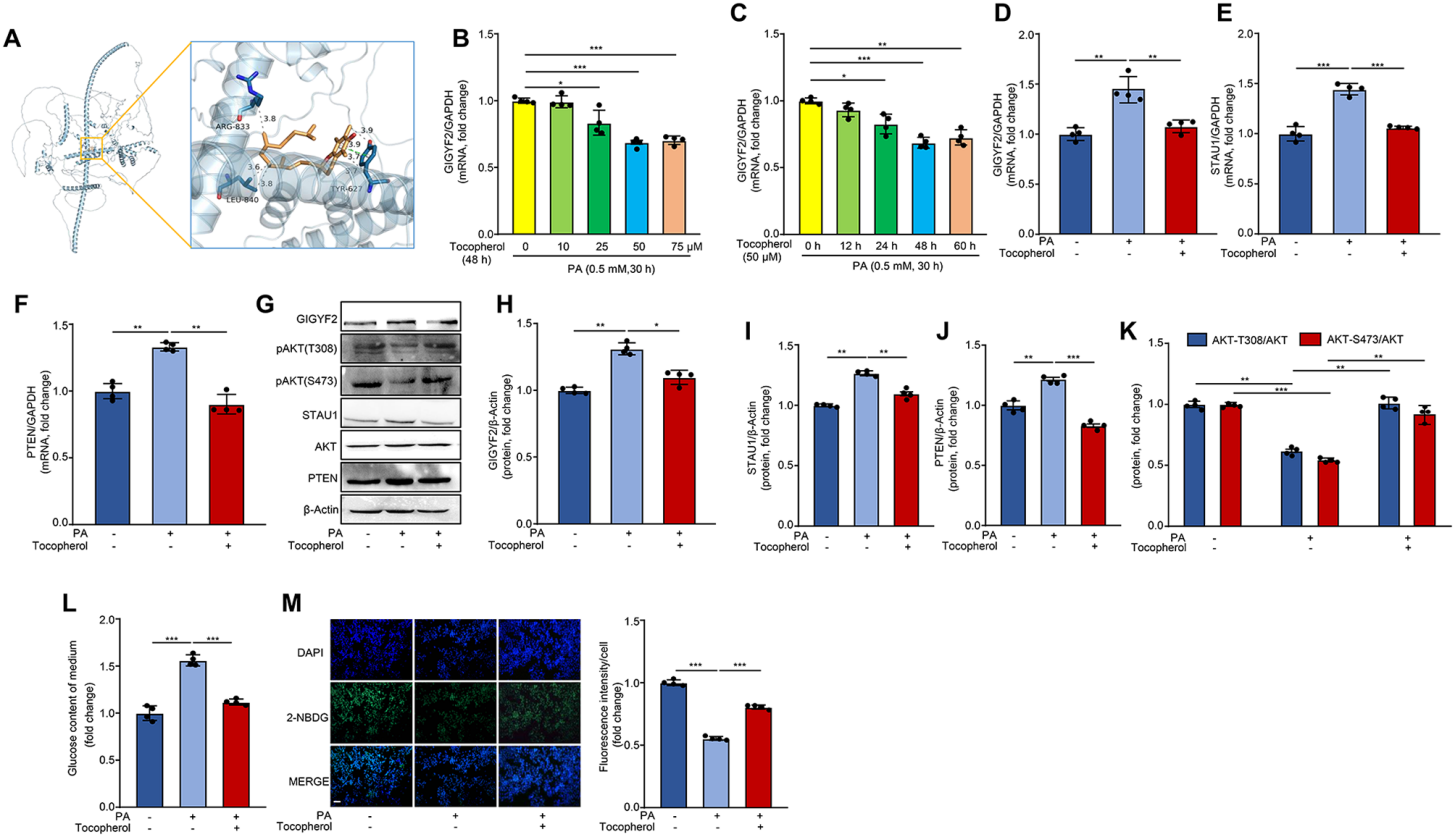
Phytopharmaceutical disruption of GIGYF2 by tocopherol ameliorates PA-induced IR in hepatocytes. (**A**) Molecular docking of tocopherol and GIGYF2 protein. qPCR analysis of the expression levels of GIGYF2 mRNA in HepG2 cells that were pre-treated with PA (0.5 mM, 30 h) and subsequently co-incubated with either (**B**) different concentrations of tocopherol (10 µM, 25 µM, 50 µM, and 75 µM) for 48 h or (**C**) 50 µM tocopherol for varying time durations (12 h, 24 h, 48 h, and 60 h). HepG2 cells were treated with PA (0.5 mM, 30 h) and then co-incubated with tocopherol (50 μm) for 48 h. (**D**) The cells were subjected to qPCR analysis of the mRNA levels of GIGYF2. (**E**) The cells were subjected to qPCR analysis of the mRNA levels of STAU1. (**F**) The cells were subjected to qPCR analysis of the mRNA levels of PTEN. (**G**) Western blotting analysis of the protein levels of GIGYF2, STAU1, PTEN and AKT phosphorylation. (**H**) Quantification of the GIGYF2 signals in (**G**). (**I**) Quantification of the STAU1 signals in (**G**). (**J**) Quantification of the PTEN signals in (**G**). (**K**) Quantification of the AKT phosphorylation signals in (**G**). (**L**) Glucose content in the medium. (**M**) Representative images of the fluorescence signaling of 2-NBDG for evaluating glucose uptake. The bar chart on the right shows the quantification of the strength signal. Scale bar = 100 μm. *n* = 4, *p < 0.05, **p < 0.01, ***p < 0.001

Next, the above findings prompted us to evaluate whether disrupting GIGYF2 with a phytopharmaceutical drug could ameliorate PA-induced IR in hepatocytes. We identified tocopherol as a potential candidate to inhibit GIGYF2 expression by searching the Pharmaco-transcriptomics Drugbank database. As shown in Fig. 7A, tocopherol exhibits strong hydrophobic forces to bind to the 840-LEU, TYR-627, and ARG-833 amino acid residues of the GIGYF2 protein. Indeed, in vitro investigation of the dose- and time-dependent effects of tocopherol on targeting GIGYF2 expression showed that 50 µM tocopherol for 48 h significantly suppressed the GIGYF2 mRNA expression in PA-treated HepG2 cells (Fig. 7B-C). Subsequently, we found that tocopherol treatment protected against PA-induced increase in mRNA and protein expression levels of GIGYF2, STAU1 and PTEN (Fig. 7D-J), and AKT inactivation (Fig. 7G, K). To further confirm the protective effect of tocopherol on glucose consumption and uptake, we measured the glucose content in the conditioned medium and the intracellular glucose uptake capacity in HepG2 cells. As shown in Fig. 7L, tocopherol is remarkable in preventing PA-induced increases in glucose content in the medium. Moreover, the PA-induced reduced glucose uptake capacity was also restored upon tocopherol treatment (Fig. 7M). These results indicate that disrupting GIGYF2 by tocopherol alleviates PA-induced hepatic IR.

### GIGYF2 knockdown alleviated high-fat diet (HFD)-induced IR in mice

**Fig. 8:**
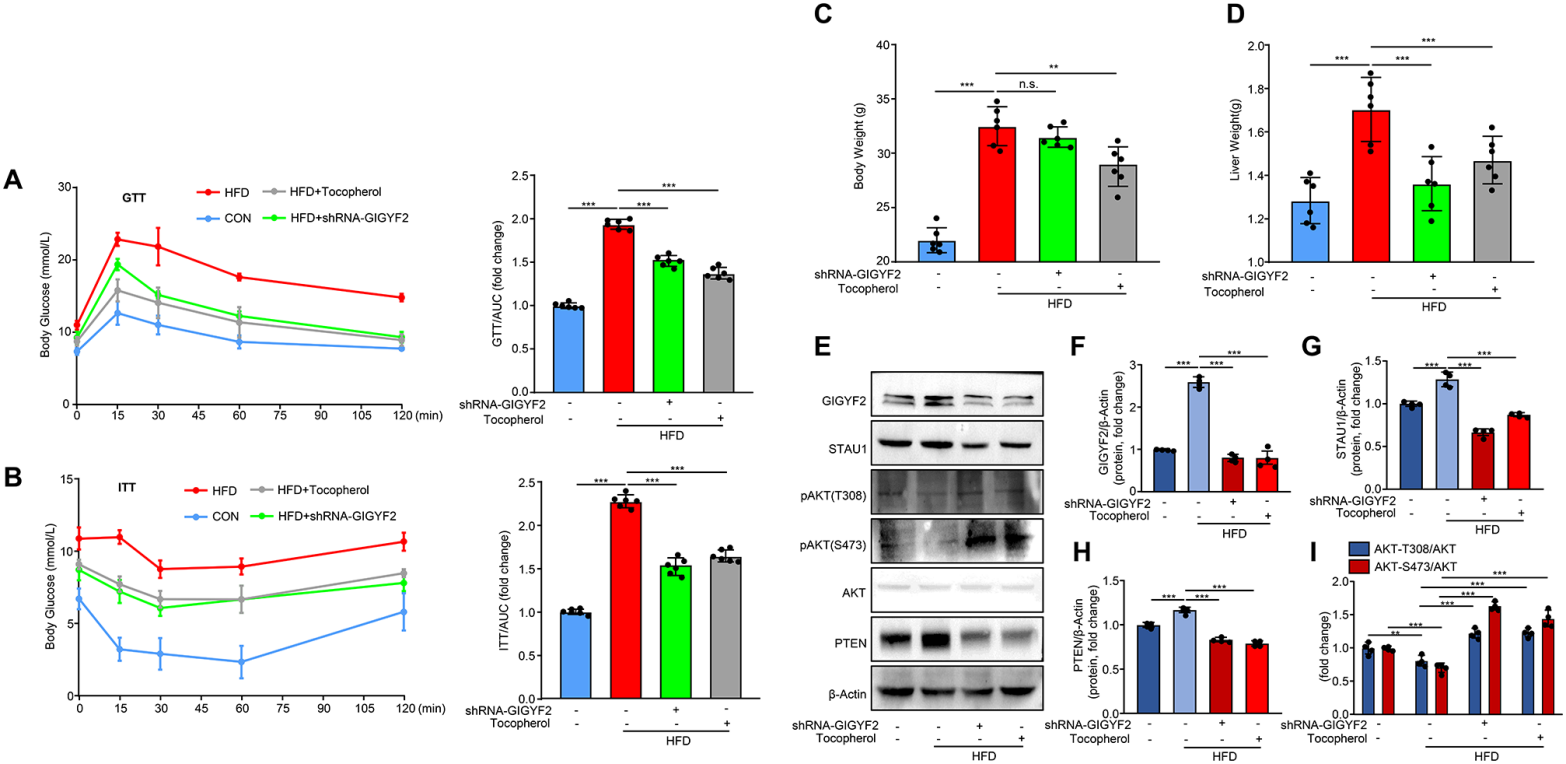
Disrupting GIGYF2 improved high-fat diet (HFD)-induced glucose intolerance and insulin sensitivity impairment in mice. Wild-type C57BL/6J mice were fed with a normal chow diet as a control group or HFD as obesity for 8 weeks, and then HFD mice were either administrated with sterile saline or tocopherol (800 mg/kg) or shRNA-GIGYF2 lentivirus for 4 weeks. (**A**) Glucose tolerance test (GTT) curve and the quantification of area under GTT curve (AUC). (**B**) Insulin tolerance test (ITT) and the quantification of area under ITT curve. (**C**) Body weight of mice. (**D**) Liver weight of mice. (**E**) Immunoblotting analysis of the protein levels of GIGYF2, STAU1, pAKT, AKT and PTEN in the liver. (**F**-**I**) Quantification of the protein levels of GIGYF2, STAU1, pAKT, AKT and PTEN in (**E**). *n* = 6. n.s.: not significant. *p < 0.05, **p < 0.01, ***p < 0.001

Based on the in vitro findings that GIGYF2 promotes IR in hepatocytes through the STAU1/PTEN/AKT axis, we therefore further investigated whether GIGYF2 mediates obesity-induced IR in obese mice fed with HFD. As expected, HFD feeding caused glucose intolerance and impaired insulin sensitivity in wild-type mice, as assessed by GTT and ITT (Fig. 8A-B). Notably, lentivirus-mediated genetic knockdown of GIGYF2 and suppression of GIGYF2 by tocopherol administration prominently improved glucose tolerance and insulin sensitivity as compared to the HFD diet alone group (Fig. 8A-B). Meanwhile, we found that the increase in body weight and liver weight provoked by HFD was attenuated by tocopherol treatment (Fig. 8C-D), whereas GIGYF2 knockdown only influenced the liver weight (Fig. 8C-D). Moreover, western analysis of mouse liver tissues revealed that protein expression levels of GIGYF2, STAU1, and PTEN were elevated and AKT phosphorylation was decreased in HFD-fed mice compared to wild-type mice, which was conversely reversed in HFD-fed mice with knockdown of GIGYF2 or tocopherol treatment (Fig. 8E-I). These in vivo results further demonstrate that disruption of GIGYF2 protects against HFD-induced IR in mice through the STAU1/PTEN axis-mediated AKT inactivation.

## Discussion

IR is characterized by damaged insulin sensitivity in the target tissues of insulin (adipose tissue, liver, and skeletal muscles) and plays a crucial role in impaired glucose homeostasis, metabolic syndrome, and T2D (Birringer et al. 2001). Whereas, the exact molecular mechanisms of obesity-induced IR in hepatic tissue are still not well elucidated. In the current study, we revealed that obesity leads to IR via upregulating GIGYF2 expression, which causes the disruption of the PI3K/AKT pathway through activation of the STAU1-PTEN signaling cascade. This study discloses a novel regulatory mechanism for GIGYF2 regulating obesity-induced IR and T2D.

PA is the most abundant saturated FFA in the diet and has been reported to induce hepatic IR by impairing cellular signaling pathways (Reynoso et al. 2003). Exposure to high levels of PA-induced pancreatic β-cell lipotoxicity has also been reported to lead to pancreatic β-cell dysfunction (Benito-Vicente et al. 2021), which increases the risk of developing diabetes. In this study, impaired glucose uptake and reduced insulin sensitivity were observed in hepatocytes subjected to prolonged and high concentrations of PA stimulation, suggesting that hepatocytes repeatedly exposed to high levels of PA may develop IR. This is consistent with previous findings that PA leads to IR in skeletal muscle cells and adipocytes (Dai et al. 2022; Sawada et al. 2012). Intriguingly, the mRNA and protein expression levels of GIGYF2 were dramatically elevated in the PA-induced hepatic IR model. Correspondingly, depleting GIGYF2 can ameliorate PA-induced IR, including decreasing the glucose content in a conditioned medium and enhancing intracellular glucose uptake. Under normal conditions without PA stimulation, overexpression of GIGYF2 per se significantly increases the glucose content and reduces glucose uptake in HepG2 cells. Moreover, in obese mice, disruption of GIGYF2 also attenuates HFD-induced glucose intolerance and IR. These results from cultured cells and mice provide conclusive in vitro and in vivo evidence for a causal role of GIGYF2 in promoting PA-induced IR. GIGYF2 is expressed ubiquitously, with abundant expression in the liver, pancreas, brain, lung, kidney, and spleen (Higashi et al. 2010). Nevertheless, the investigation into how GIGYF2 regulates obesity-related IR remains unclear. Previous studies have identified that the aberrant expression of GIGYF2 can impair cognitive function in diabetic mice by altering insulin-like growth factor signaling (Blum et al. 2014; Giovannone et al. 2009). Further investigation remains warranted to determine whether GIGYF2 mediating PA-associated lipid homeostasis contributes to obesity-related IR. Here, we demonstrate that dysregulated expression of GIGYF2 is involved in the modulation of obesity-induced IR.

Considerable evidence supports the close association between dysfunction in the PI3K/AKT signaling pathway and IR in diverse tissues throughout the body (Zhang et al. 2019). The tumor suppressor PTEN serves as a negative regulator of the PI3K/AKT signaling pathway, and inhibition of PTEN activity can activate the AKT signal transduction pathway (Bu et al. 2021; Driessen et al. 2016; Wishart and Dixon 2002; Yue et al. 2014). Studies have indicated that PTEN plays a role in regulating various metabolic functions across a diverse array of tissues. These functions encompass insulin sensitivity, glucose homeostasis, energy balance, obesity, and the levels of metabolic hormones (Li et al. 2020). The dysregulated PTEN expression in T2D leads to impaired insulin signaling and promotes IR in the pathogenesis of T2D (Li et al. 2020). Strikingly, here we found that overexpression of GIGYF2 alone promoted PTEN expression, inhibited the PI3K/AKT pathway, and impaired intracellular glucose uptake. Meanwhile, in PA-induced hepatocyte IR, silencing of GIGYF2 significantly down-regulated PTEN expression, and prevented the inactivation of PI3K/AKT signaling, along with ameliorating IR. Consistently, silencing PTEN inhibits the PI3K/AKT pathway and is also able to ameliorate GIGYF2-induced IR in hepatocytes, for example, reduces glucose content of the conditioned medium and enhances intracellular glucose uptake. These results for the first time illustrate the pivotal role of GIGYF2 in regulating PTEN-mediated inactivation of the PI3K/AKT pathway, leading to the development of IR and T2D in the context of obesity. Consistent with our data, Yang et al. found that GIGYF2 acts as a tumor suppressor to negatively regulate AKT/Bax/Caspase-3 signaling, and thus control cell death/survival in glioma cells (Yang et al. 2021). In our recent study, we also found that GIGYF2 activates the mTORC1-S6K1 signaling pathway via recruiting mTORC1 to the lysosomal membrane, leading to endothelial cell senescence, dysfunction, and vascular aging (Niu et al. 2023). mTORC1 can function as a negative feedback modulator to suppress mTORC2 activity through S6K1-mediated phosphorylation and degradation of insulin receptor substrate1/2 (Sun et al. 2023). As such, GIGYF2 may negatively orchestrate AKT activity via activating the mTORC1-S6K1 signaling cascade in hepatocytes. Whereas, in this regard, whether this is true still warrants further investigation.

An important novel finding of our study is to disclose that obesity promotes PTEN expression through the GIGYF2-STAU1 signaling cascade, which results in the inactivation of the PI3K/AKT pathway, ultimately contributing to the pathogenies of IR. STAU1, a prototypical double-stranded RNA (dsRNA) binding protein, participates in numerous biological processes, including cell proliferation (Ghram et al. 2020), apoptosis (Gandelman et al. 2020), migration (Ramasamy et al. 2006), differentiation (Gautrey et al. 2005), autophagy (Paul et al. 2021), and stress responses (Thomas et al. 2009). Previous transcriptome analyses have shown that STAU1 was markedly downregulated in the GIGYF2-deficient senescent endothelial cells (Niu et al. 2023). Intriguingly, we found that STAU1 expression was dramatically elevated in a PA-induced IR model of hepatocytes. Overexpressing GIGYF2 significantly promoted STAU1 expression, whereas GIGYF2 depletion significantly suppressed PA-induced upregulation of STAU1. In contrast, GIGYF2 expression was not affected by STAU1 knockdown, indicating that there is no crosstalk between GIGYF2 and STAU1. These findings suggest that GIGYF2 might trigger hepatocyte IR via the upregulation of STAU1 expression. This is supported by the fact that STAU1 knockdown not only blocks PTEN-mediated inactivation of PI3K/AKT signaling evoked by GIGYF2 but also ameliorates GIGYF2-induced hepatic IR. Meanwhile, we further provide evidence that overexpressing STAU1 per se markedly enhances PTEN-mediated inactivation of PI3K/AKT signaling and induces IR features in HepG2 cells. In an animal model, liver tissues from high-fat diet (HFD) mice with GIGYF2 knockdown or those treated with tocopherol exhibit diminished expression of GIGYF2, STAU1, and PTEN, accompanied by increased phosphorylation of AKT. Consistent with our data, sustained STAU1 expression in postnatal skeletal muscle mediates PTEN expression through indirect transcription and direct post-transcriptional events, which negatively regulates the PI3K/AKT signaling pathway, leading to a myopathy characterized by significant morphological and functional deficits (Crawford Parks et al. 2017). In renal cancer, lncTCL6 attenuates cancer progression by suppressing Src-AKT driven metastatic pathway via STAU1-mediated Src mRNA decay (Kulkarni et al. 2021). However, the question of how GIGYF2 upregulates STAU1 remains unsolved in this study. Our previous study revealed that GIGYF2 could serve as an RBP to modulate the stability of STAU1 mRNA in vascular endothelial cells (Niu et al. 2023), hence we speculate that GIGYF2 may also promote STAU1 expression by regulating the STAU1 mRNA stability.

Next, we further answered the question that how STUA1 regulates PTEN-mediated inactivation of the PI3K/AKT pathway. STAU1 has been identified to play a crucial role in RNA localization (Hassine et al. 2020), splicing (Cox et al. 2016), stability (Xu et al. 2015), translation (Dugre-Brisson et al. 2005), and decay (Park and Maquat 2013). A previous study by Sugimoto et al. identified a Staufen1-binding secondary structure in the 3’UTR of PTEN mRNA (Sugimoto et al. 2015). In neuromuscular disorders, STUA1 was reported to interact with endogenous PTEN mRNA and enhance PTEN expression via the 3’UTR (Crawford Parks et al. 2017). In this study, we further found that STUA1 binds to PTEN mRNA and upregulates PTEN expression via enhancing its mRNA stability, which is evidenced by the fact that silencing STUA1 attenuates PTEN mRNA decay. Moreover, STAU1, an RNA-binding protein (RBP) featuring UUUAA motifs, binds to the 3’UTR region of PTEN (chr7: 87865188–87865212), regulating PTEN mRNA expression. This is evidenced by the lack of interaction between STAU1 and mutated binding sites, and the elevated expression of PTEN mRNA upon overexpression of the STAU1 gene. Silencing PTEN markedly impeded the inactivation of the PI3K/AKT pathway and IR caused by the overexpression of STAU1. Notably, we showed that the knockdown of PTEN fails to blockade the upregulation of STAU1 provoked by PA stimulation. There is no crosstalk or feedback between STAU1 and PTEN. These data point to a role for STAU1 in modulating obesity-related IR by enhancing the stability of PTEN mRNA through its binding to the PTEN 3’UTR, consequently inhibiting the PI3K/AKT signaling pathway.

Finally, the above results prompted us to evaluate whether disrupting GIGYF2 by chemical inhibitors or genetic deficiency improves PA-induced hepatocyte IR. The human body has large amounts of the fat-soluble vitamin tocopherol, which is essential for carrying out daily tasks and found in many different tissues and organs of the body. Tocopherol is known to be deficient in diabetes, and it provides a protective effect against the development of diabetes in humans (Jain 2012; Shahidi and de Camargo 2016). Whereas, the action mechanism of tocopherol still remains mysterious. The pharmaco-transcriptomics Drugbank database shows that tocopherols are potential candidates for down-regulation of GIGYF2 expression. Here, we reveal that tocopherol prevents the upregulation of GIGYF2, STAU1, and PTEN-mediated PI3K/AKT inactivation induced by PA treatment, along with ameliorating PA-induced IR in hepatocytes. In obese mice with IR, we confirm that tocopherol shows comparable effects with GIGYF2 knockdown to markedly ameliorate glucose tolerance and insulin sensitivity. These results substantially support the notion that disrupting GIGYF2 could improve obesity-related IR.

In summary, our study disclosed a novel function for the gene GIGYF2 in obesity-induced IR. Upregulation of GIGYF2 promotes STAU1 expression as an RBP, which enhances PTEN mRNA stabilization to increase PTEN expression, thereby inhibiting the PI3K/AKT signaling pathway, ultimately contributing to impaired insulin activity. Targeting GIGYF2 may represent a promising therapeutic approach for the treatment of IR-related diabetes.

### Electronic supplementary material

Below is the link to the electronic supplementary material.

Supplementary Material 1

## Data Availability

No datasets were generated or analysed during the current study.
