# Supplementary material for: RNA-binding protein GIGYF2 orchestrates hepatic insulin resistance through STAU1/PTEN-mediated disruption of the PI3K/AKT signaling cascade
Source: Mol Med. 2024 Aug 13;30:124. doi: 10.1186/s10020-024-00889-6 (PMC11323356; doi:10.1186/s10020-024-00889-6)
Supplement: Supplementary file 1 — Supplementary Material 1 [file 10020_2024_889_MOESM1_ESM.pdf]

Supplemental Figures

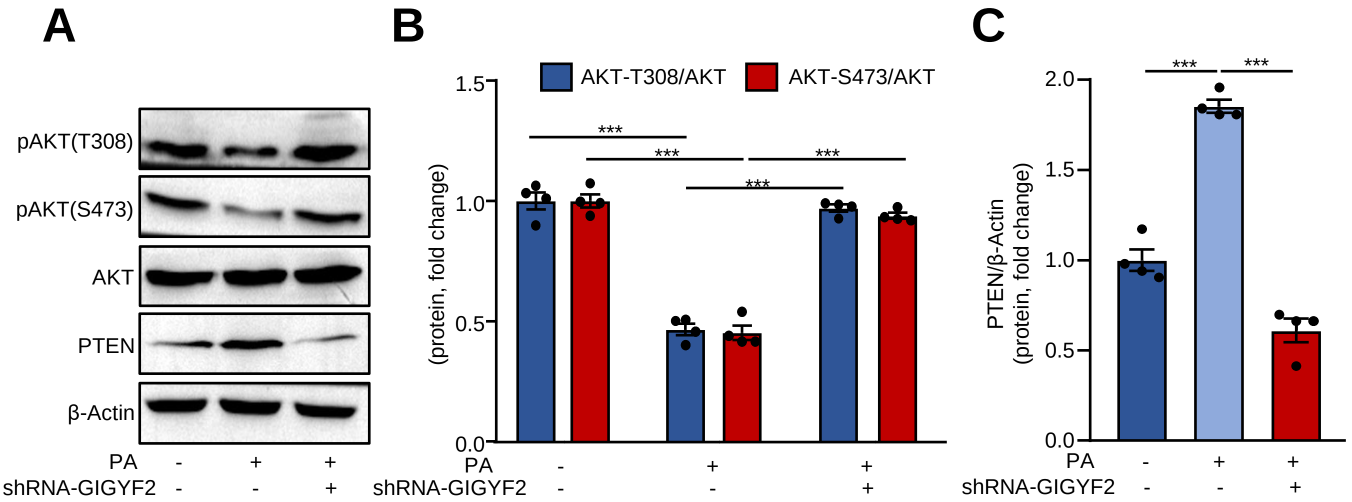

Supplementary Fig. 1: Silencing GIGYF2 ameliorates PA-induced PTEN elevation, and AKT inactivation in HepG2 cells. HepG2 cells were transfected with knock-down lentivirus shRNA-GIGYF2, GIGYF2 was silenced, and then treated with PA for 30h. **(A)** Western blotting was performed to analyze the protein expression levels of PTEN, AKT-Thr308 and AKT-Ser473. **(B)** Quantification of relative levels of Thr308 and Ser473. **(C)** Quantification of the relative levels of PTEN. n = 4, $^{*}p < 0.05$, $^{**}p < 0.01$, $^{***}p < 0.001$.

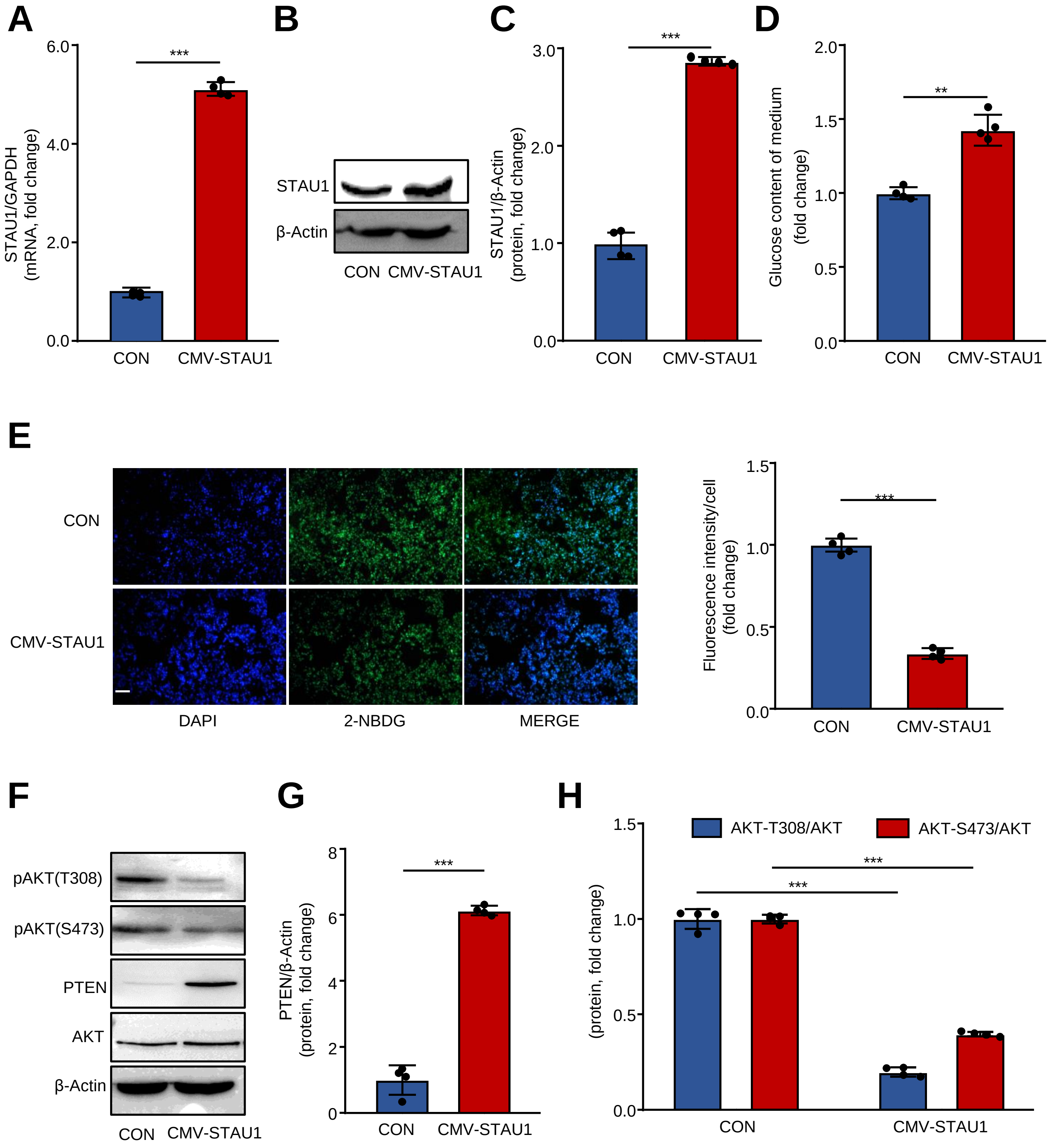

Supplementary Fig. 2: Overexpression of STAU1 exacerbates insulin resistance in HepG2 cells. HepG2 cells were transduced for two days with overexpressed CMV empty lentivirus as control (CON) or CMV-STAU1. The cells were subjected to **(A)** qPCR analysis of the STAU1 mRNA levels and **(B)** western blotting analysis of STAU1 protein levels with their quantification **(C)**. **(D)** Measurement of glucose content of the medium. **(E)** Fluorescent staining analysis of 2-NBDG uptake. **(F)** Western blotting analysis of the expression levels of PTEN, AKT, AKT-Thr308, and AKT-Ser473 in CON and STAU1 groups. **(G)** Represents quantification of the relative levels of PTEN protein normalized to β-Actin. **(H)** Quantification of relative levels of AKT-Thr308 and AKT-Ser473 proteins normalized to AKT. Scale = 100 μm. n = 4, $^{**}p < 0.01$, $^{***}p < 0.001$.

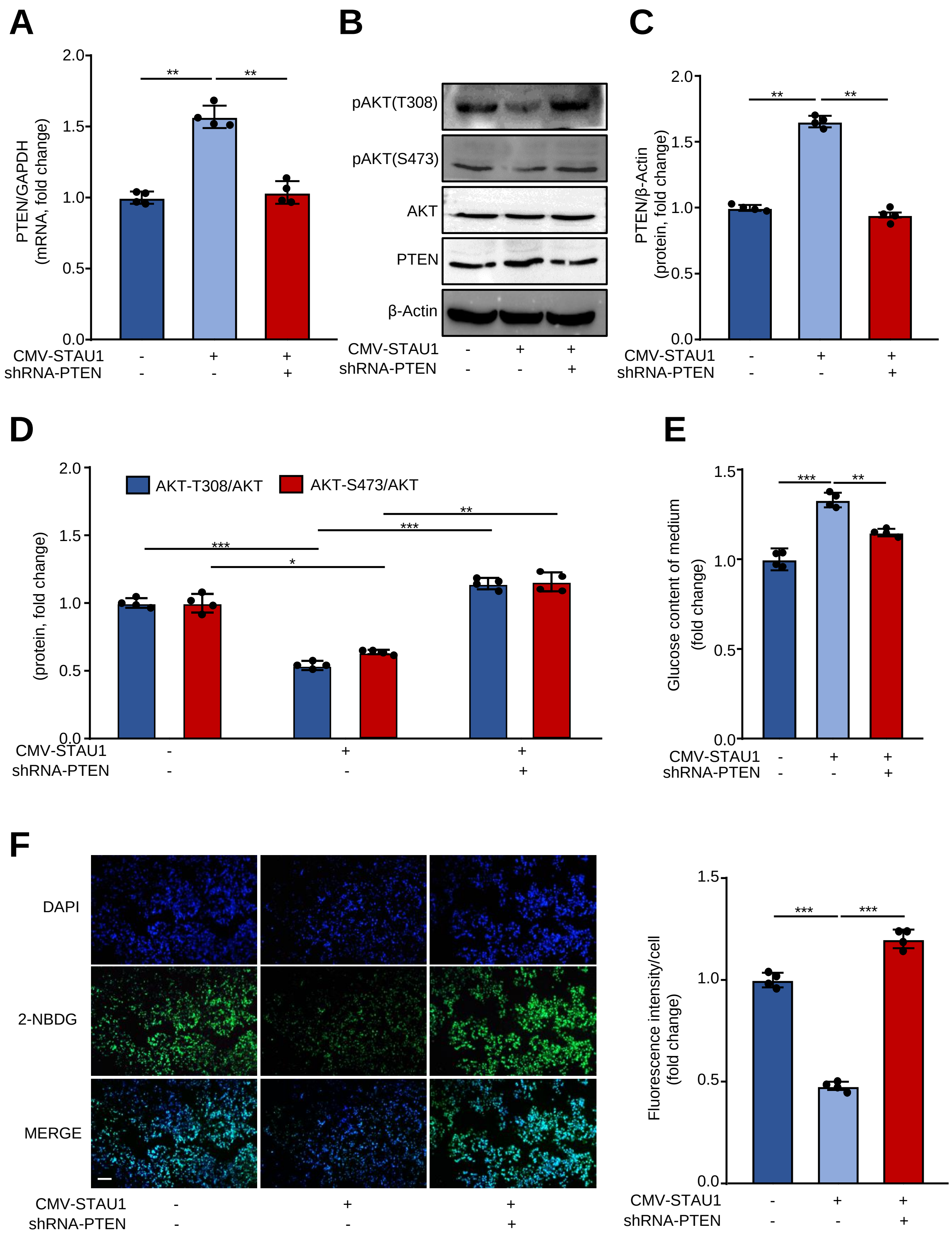

Supplementary Fig. 3: PTEN deficiency mitigates STAU1-induced IR and AKT activation. HepG2 cells were transduced with lentivirus CMV-empty as control or CMV-STAU1. 24 h post-transduction, cells were transduced with lentivirus-mediated PTEN shRNA for silencing PTEN. 48 h post-transduction, the cells were subjected to **(A)** qPCR analysis of the mRNA levels of PTEN. **(B)** Western blotting analysis of the protein levels of STAU1, PTEN and AKT phosphorylation. **(C)** Quantification of the PTEN signals in (B). **(D)** Quantification of the AKT phosphorylation signals in (B). **(E)** The glucose content of the medium. **(F)** Representative images of the fluorescence signaling of 2-NBDG for evaluating glucose uptake. Scale bar = 100 μm. n = 4, *p < 0.05, **p < 0.01, ***p < 0.001.
